# Single-cell transcriptomics reveals a compartmentalized antiviral interferon response in the nasal epithelium of mice

**DOI:** 10.1128/jvi.01413-24

**Published:** 2025-02-04

**Authors:** Xuefei Wang, Meng Dong, Xinchao Wu, Daniel Schnepf, Julia Thiel, Wenfei Sun, Christian Wolfrum, Sisi Li, Wenfei Jin, Peter Staeheli, Liang Ye

**Affiliations:** 1 Department of Immunology, International Cancer Center, Shenzhen University Medical School 481870 , Shenzhen, China; 2 Shenzhen Key Laboratory of Gene Regulation, Department of Systems Biology School of Life Sciences, Southern University of Science and Technology 255310 , Shenzhen, China; 3 Dr. Margarete Fischer-Bosch Institute of Clinical Pharmacology and University of Tübingen 40011 , Stuttgart, Germany; 4 Institute of Virology, Medical Center University of Freiburg 14879 , Freiburg, Germany; 5 Immunoregulation Laboratory, The Francis Crick Institute, London, United Kingdom; 6 Institute of Food, Nutrition and Health, ETH Zurich, Schwerzenbach, Switzerland; 7 CAS Key Laboratory of Computational Biology, Shanghai Institute of Nutrition and Health, University of Chinese Academy of Sciences, Chinese Academy of Sciences 26443 , Shanghai, China; St. Jude Children's Research Hospital, Memphis, Tennessee, USA

**Keywords:** interferons, respiratory tract, SARS-CoV-2

## Abstract

Type III interferons (IFNs) primarily act on epithelial cells and protect against virus infection of the mucosa, whereas type I IFNs act more systemically. To date, it has been unknown which epithelial subtypes in the upper airways, the primary site for initial infection for most respiratory viruses, primarily rely on type III IFN or type I IFNs for antiviral protection. To address this question, we performed a single-cell transcriptomics analysis of the epithelial IFN-mediated response focusing on the upper airways of mice. This work identified nine distinct cell types derived from the olfactory epithelium and thirteen distinct cell types from the respiratory epithelium. Interestingly, type I IFNs induced a stronger antiviral transcriptional response than type III IFN in respiratory epithelial cells, whereas in olfactory epithelial cells, including sustentacular (SUS) and Bowman’s gland cells (BGC), type III IFN was more dominant compared to type I IFN. SUS and BGC, which provide structural support and maintain the integrity of olfactory sensory neurons, were highly susceptible to infection with a mouse-adapted variant of severe acute respiratory syndrome coronavirus 2 (SARS-CoV-2 MA20) but were protected against infection if the animals were prophylactically treated with type III IFN. These findings demonstrate a high degree of cell type heterogeneity in terms of interferon-mediated antiviral responses and reveal a potent role for type III IFNs in protecting the olfactory epithelium.

**IMPORTANCE:**

SARS-CoV-2 infects SUS and BGC in the olfactory epithelium, causing an impairment of structural support and integrity of olfactory sensory neurons that can result in severe olfactory dysfunctions. We observed an unexpected compartmentalization of the IFN-mediated transcriptional response within the airway epithelium, and we found that olfactory epithelial cells preferentially respond to type III IFN, which resulted in robust antiviral protection of SUS and BGC. Given the proximity of the olfactory epithelium to the central nervous system, we hypothesize that evolution favored a type III IFN-biased antiviral immune response in this tissue to limit inflammatory responses in the brain. Cell type-specific antiviral responses in the upper airways, triggered by the different types of IFNs, should be investigated in more detail and carefully taken into consideration during the development of IFN-based antivirals for clinical use.

## INTRODUCTION

Type I and III interferons (IFNs) represent a first line of defense against virus infection and have attracted plenty of attention during the SARS-CoV-2 pandemic (1, 2). Type I IFNs (IFN-α/β) bind to their heterodimeric receptor complex consisting of IFNAR1 and IFNAR2, which are expressed on almost all nucleated cells, to induce a systemic antiviral response. Type III IFNs (IFN-λ) bind a distinct receptor complex consisting of the specific IFNLR1 receptor subunit, expressed by epithelial cells and some specific immune cells, to form a heterodimeric complex with the ubiquitously expressed IL10Rβ subunit (3). Although both IFN-α/β and IFN-λ activate a highly overlapping Janus kinase (JAK)-signal transducer of transcription (STAT) pathway to upregulate the transcription of antiviral IFN-stimulated genes (ISGs), a nonredundant antiviral role of IFN-λ became apparent at mucosal barriers, including the respiratory tract (3). Previous studies showed that IFN-λ compared to IFN-α/β is much more potent in limiting respiratory virus transmission and dissemination from the upper airways to the lungs (3–5). Furthermore, predominantly IFN-λ was found to drive the expression of protective ISGs in the upper respiratory tract of patients with mild COVID-19 disease (6), confirming the indispensable role of IFN-λ in the upper airways of humans. Interestingly, IFN-λ, but not IFN-α/β, selectively acts on microfold (M) cells in the upper airways to enhance adaptive mucosal antiviral immunity by upregulating the expression of thymic stromal lymphopoietin (TSLP) (7, 8), further demonstrating important nonredundant biological functions of the observed cell type-specific heterogeneity of the IFN response in the upper airway epithelium. Although both IFN-λ and IFN-α/β could reduce the viral load in COVID-19 patients and decreased the incidence of hospitalization (9–11), it remains unclear whether and how both types of IFN might regulate the antiviral response in upper airway epithelial cells, which results in protection against virus infection and olfactory dysfunction.

Severe acute respiratory syndrome coronavirus 2 (SARS-CoV-2) is a pandemic virus causing coronavirus disease 2019 (COVID-19), which initially affects the upper respiratory tract (URT). The viral spike protein binds its host receptor angiotensin-converting enzyme 2 (ACE2) on the nasal epithelium, and viral entry is mediated after the proteolytic priming cleavage by furin, followed by the action of transmembrane serine protease (TMPRSS2) on the cell surface or other cysteine proteases in the endosomal compartment (12–15). Although SARS-CoV-2 infection frequently results in severe airway and pulmonary symptoms, many patients also exhibit extra-respiratory symptoms with variable severity, including olfactory or gustatory dysfunction and even neurological complications (16–19). Of note, olfactory dysfunction is one of the most common symptoms in COVID-19 and is considered to be a predictive factor for SARS-CoV-2 infection (17, 19–21). Recent studies aimed at determining how SARS-CoV-2 affects the olfactory system demonstrated that ACE2 and TMPRSS2 are abundantly expressed in human and mouse olfactory systems, which may facilitate SARS-CoV-2 entry (19, 21–23). A primary target of SARS-CoV-2 in the olfactory epithelium are sustentacular cells (SUS) that provide structural support and maintain the integrity of olfactory sensory neurons (OSN) (19–21, 24). SARS-CoV-2 infection of SUS induces olfactory impairment, which might be a result of structural support, odorant receptor gene downregulation on OSN, loss of OSN cilia, and inflammatory cell migration into the olfactory epithelium (20, 25). While hypotheses about underlying mechanisms of COVID-19 olfactory disorders are gradually emerging, many details remain to be clarified.

In this study, we employed single-cell RNA sequencing (scRNA-seq) to delineate mouse nasal epithelial cell heterogeneity and to investigate how type I and type III IFNs elicit their specific antiviral programs in the upper airways. We found that IFN-λ induced a strong antiviral response predominantly in the olfactory epithelium, while IFN-α mainly acted on the respiratory epithelium. SUS and Bowman’s gland cells (BGC) were identified as major host cells of SARS-CoV-2 in the olfactory epithelium, and we found that IFN-λ effectively blocked SARS-CoV-2 infection of these cells. These results highlight significant differences between IFN-α/β- and IFN-λ-mediated cell type-specific transcriptional responses in the upper airways and indicate that IFN-λ could play a decisive role in preventing olfactory dysfunctions during viral infections.

## RESULTS

### Landscape of the mouse nasal epithelium

To profile transcriptional changes in upper airway epithelial cells, type I or type III IFN was applied to the nostrils of mice. At 5 hours post-treatment, the animals were killed, and the snouts were dissected mechanically, followed by enzymatic digestion before CD45^-^ cells were isolated for scRNA-seq. Due to the small tissue size, we pooled the material from 15 mice of each group to retrieve enough cells for subsequent scRNA-seq.

**Fig 1:**
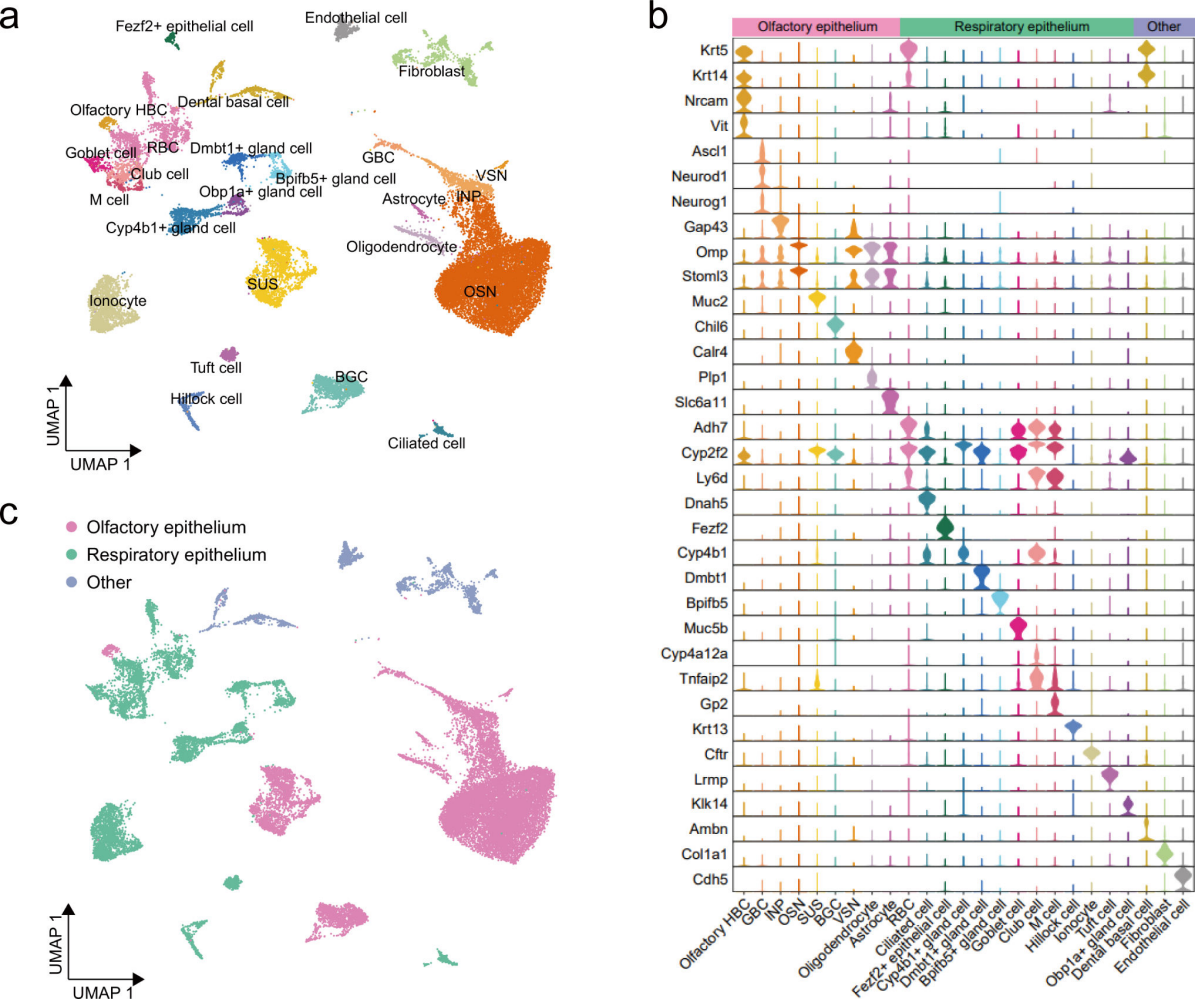
Landscape of mouse upper airway epithelial cells. (a) UMAP was used to visualize a total of 29,470 cells from mouse snout tissue. Using marker genes, 25 cell types could be identified. Each point represents one cell, and each cell type was color-coded. (b) Violin plot of marker genes for each cell type. One or more marker genes of each identified cell type were presented. Olfactory HBC, olfactory horizontal basal cell; GBC, global basal cell; INP, intermediate neural progenitors; OSN, olfactory sensory neuron; SUS, sustentacular cell; BGC, Bowman’s gland cell; VSN, vomeronasal sensory neuron; RBC, respiratory basal cell; M cell, microfold cell. (c) UMAP visualization of all cells colored by three large categories, namely, olfactory epithelium, respiratory epithelium, and others.

We first constructed a comprehensive landscape based on scRNA-seq data from 29,470 cells derived from IFN-α-, IFN-λ-treated, and naïve mice as no batch effects were observed across the different groups, and all identified cell types were consistently present in each group (Fig. 1A). This analysis identified nine distinct olfactory epithelium cell types, namely, olfactory horizontal basal cells (HBC) (*Krt5* and *Nrcam*), globose basal cells (GBC) (*Ascl1* and *Neurod1*), intermediate neural progenitors (INP) (*Gap43* and *Omp*), olfactory sensory neurons (OSN) (*Omp* and *Stoml3*), sustentacular cells (SUS) (*Krt18* and *Muc2*), bowman’s gland cells (BGC) (*Sox9* and *Chil6*), vomeronasal sensory neurons (VSN) (*Calr4* and *Gng2*), oligodendrocytes (*Plp1* and *Fabp7*), and astrocytes (*Slc6a11* and *Gria2*) (19, 21, 23, 26) (Fig. 1b and c; Fig. S1). Furthermore, we identified 13 cell types from the respiratory epithelium in our data set, including respiratory basal cells (RBC) (*Krt5* and *Adh7*), ciliated cells (*Dnah5* and *Foxj1*), goblet cells (*Muc5b* and *Muc5ac*), club cells (*Cyp2f2* and *Cyp4a12a*), microfold (M) cells (*Gp2* and *Cp*), hillock cells (*Krt13* and *Asprv1*), ionocyte (*Cftr* and *Coch*), tuft cells (*Lrmp* and *Trpm5*), *Fezf2*^+^ epithelial cells (*Fezf2*), and four cell types expressed the acinar or duct cell markers *Aqp5* or *Bhlha15*, namely, *Cyp4b1*^+^ gland cells, *Dmbt1*^+^ gland cells, *Bpifb5*^+^ gland cells, and *Obp1a*^+^ gland cells (19, 21, 23, 26) (Fig. 1a and b; Fig. S1). Due to technical limitations in tissue sampling, three non-UTR epithelial cell types, namely, fibroblasts (*Col1a1* and *Lum*), endothelial cells (*Cdh5* and *Egfl7*), and dental basal cells (*Krt5*, *Ambn,* and *Amtn*) (27, 28), were detected but not further investigated because they were outside the focus of our investigation (Fig. 1a and b; Fig. S1). Thus, our experimental approach enabled a simultaneous observation of a wide spectrum of different cell types from the nasal epithelium of mice.

### IFN-λ preferentially targets the olfactory epithelium, whereas IFN-α primarily acts on the respiratory epithelium

**Fig 2:**
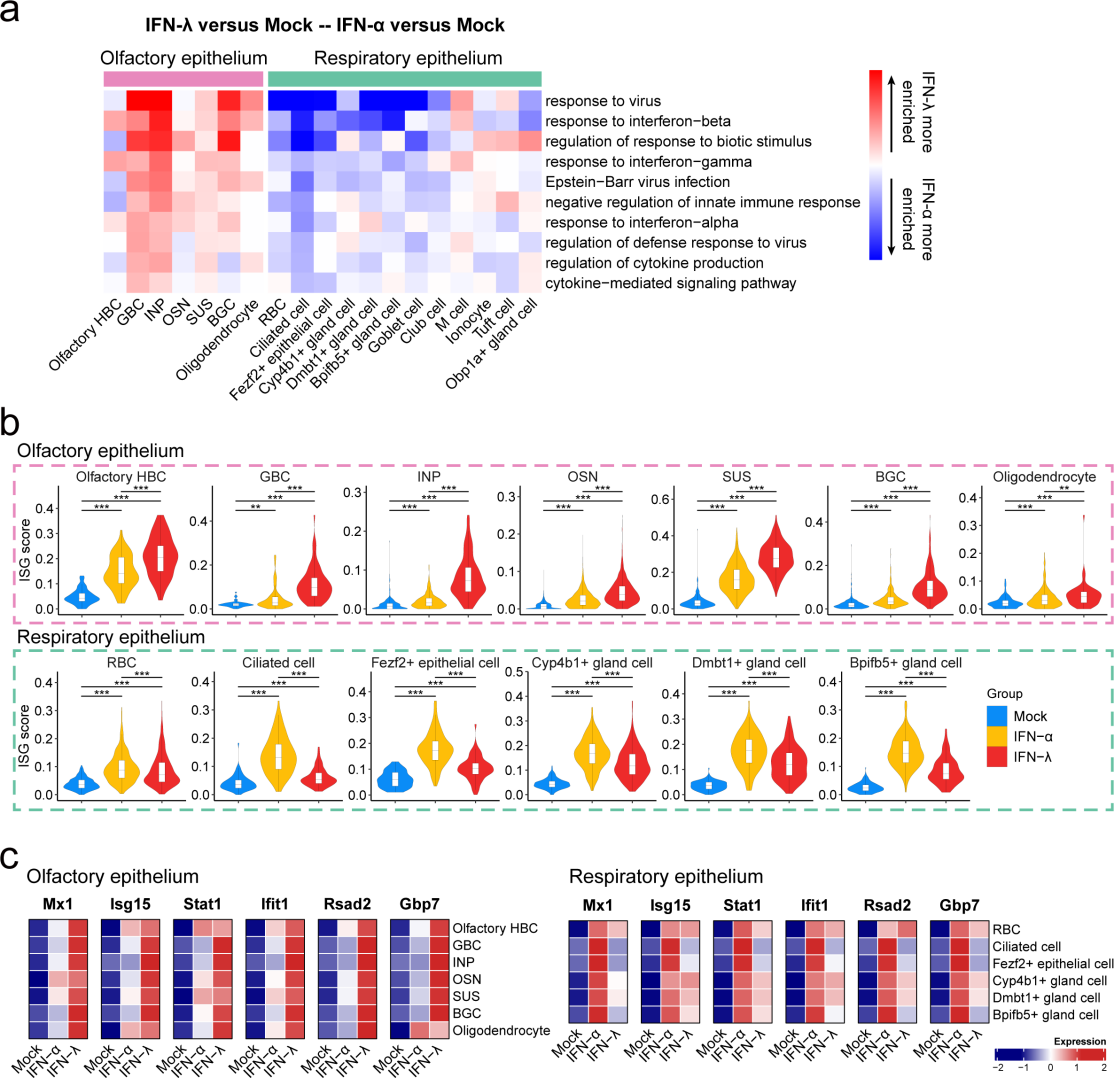
IFN-λ triggers strong antiviral responses in the olfactory epithelium, whereas IFN-α preferentially targets respiratory epithelial cells. (a) The difference in the top 10 common GO terms in each cell type between the IFN-α group and the IFN-λ group compared to the mock group. Each row and column represent the GO term and cell type, respectively. The color represents the difference in -Log_10_ (*P* value) for the top 10 common GO term enrichments of highly expressed genes in the IFN-α group or the IFN-λ group compared to the mock group. This difference is calculated by subtracting the -Log_10_ (*P* value) of IFN-λ versus mock from that of IFN-α versus mock. Red indicates greater enrichment for the IFN-λ group, while blue indicates greater enrichment for the IFN-α group. (b) Violin plot of interferon-stimulated gene (ISG) score in olfactory and respiratory epithelial cell types. The ISG score was calculated by the AUCell package based on 60 canonical ISGs. The statistical differences in the ISG score among the three groups were calculated by the Wilcoxon test (two-sided). *P < 0.05; **P < 0.01; ***P < 0.001. (c) Scaled expression of *Mx1*, *Isg15*, *Stat1*, *Ifit1*, *Rsad2,* and *Gbp7* on olfactory epithelial cells (left panel) and respiratory epithelial cells (right panel).

Most cell populations in the nasal epithelium showed no increased effect score at 5 hours post IFN-α or IFN-λ treatment (Fig. S2a), although slightly enhanced values were observed in SUS, ionocytes, Bpifb5^+^ gland cells, and OSN (Fig. S2b and c). To identify cell type-specific responses, we compared differentially expressed genes and signaling pathways in each nasal epithelial cell type from IFN-α- and IFN-λ-treated mice. Gene ontology (GO) term enrichment analysis revealed that the majority of olfactory epithelial cells, including GBC, INP, OSN, SUS, and BGC, responded more strongly to IFN-λ than IFN-α by upregulation of genes that are associated with “response to virus,” “response to interferon,” and “interferon signaling” pathways (Fig. 2a; Fig. S3a). In contrast, most respiratory epithelial cell types, including RBCs, ciliated cells, Fezf2^+^ epithelial cells, Cy4b1^+^ gland cells, Dmbt1^+^ gland cells, Bpifb5^+^ gland cells, and ionocytes, responded primarily to IFN-α rather than IFN-λ. Interestingly, M cells within the respiratory epithelium cluster were exceptional in their biased IFN response, and they responded more strongly to IFN-λ instead of IFN-α (Fig. 2a and Fig. S4), which is in line with previous observations (7). Gene set enrichment analysis (GSEA) confirmed enhanced enrichment of genes associated with “response to virus” in olfactory epithelial cells treated with IFN-λ and in respiratory epithelial cells treated with IFN-α (Fig. S3b).

Interferons protect against viral infection by upregulating the expression levels of hundreds of IFN-stimulated genes (ISGs), of which many have direct antiviral activity (29). To evaluate the cell type-specific antiviral potential of IFN-α and IFN-λ, we compared the expressions of 60 canonical ISGs in the various epithelial cell subtypes of treated mice. In olfactory epithelial cells, the highest ISG score was typically observed after IFN-λ treatment, whereas in respiratory epithelial cells, the highest ISG score was usually seen after IFN-α treatment (Fig. 2b; Fig. S4). Consistent with these findings, in the olfactory epithelium, IFN-λ upregulated the expression levels of ISGs associated with virus defense (such as *Mx1*, *Isg15*, *Stat1*, *Ifit1, Rsas2,* and *Gbp7*) more strongly than IFN-α, whereas in the respiratory epithelium, IFN-α triggered the expression of these ISGs more strongly than IFN-λ (Fig. 2c). Overall, these findings illustrate a high degree of cell type-specific heterogeneity of type I and type III IFN-mediated responses in the respective epithelial cell types lining the upper airways, with IFN-λ preferentially acting on the olfactory epithelium and IFN-α mainly acting in the respiratory epithelium.

### IFN-λ induces a stronger antiviral response than IFN-α in SUS and BGC

**Fig 3:**
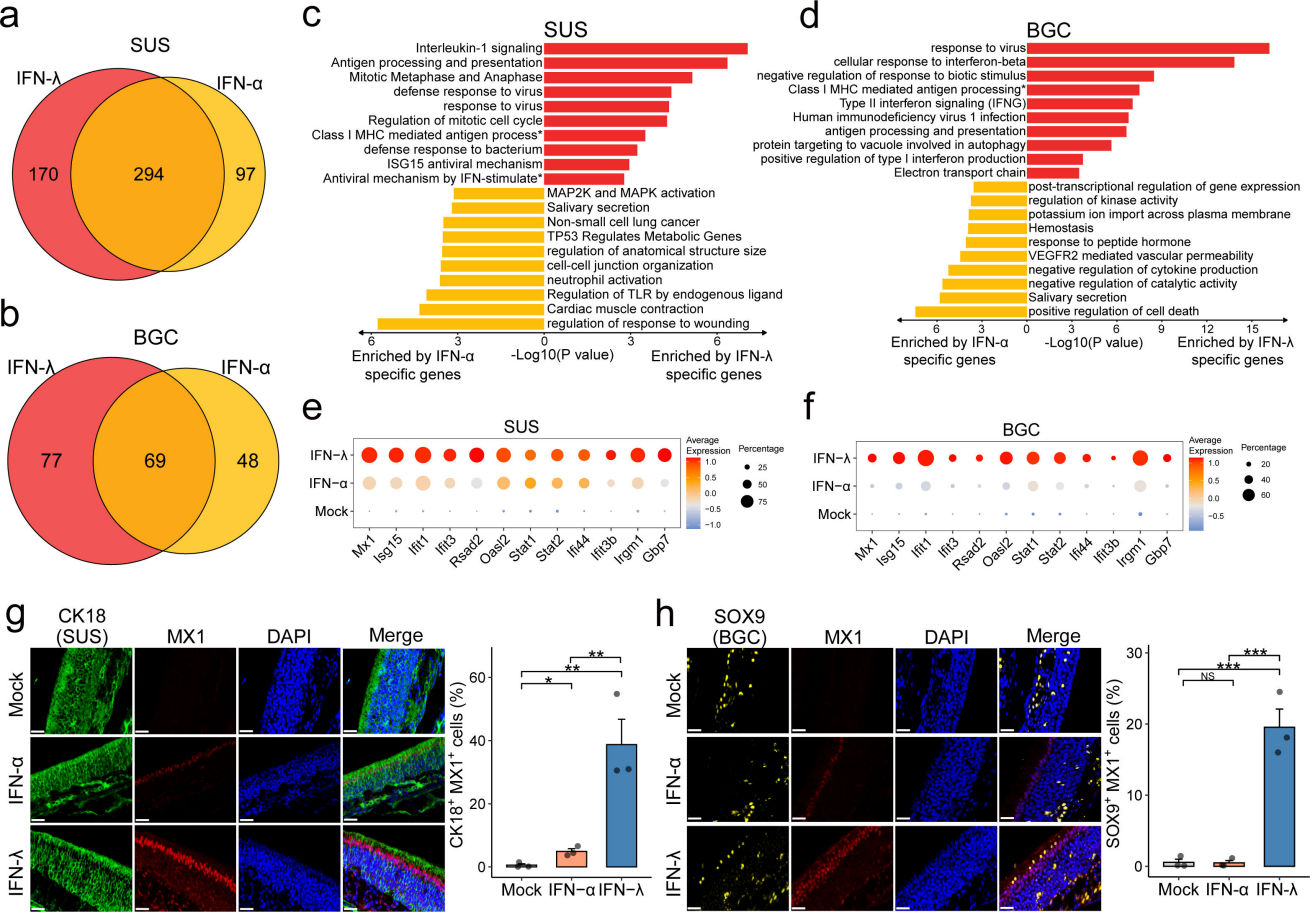
Preferential IFN-λ responses of SUS and BGC. (a, b) Venn diagram showing the overlap of highly expressed genes between IFN-λ versus mock or IFN-α versus mock in SUS (a) or BGC (b). The red region represents highly expressed genes only in the IFN-λ group; the yellow region represents highly expressed genes only in the IFN-α group; and the orange region represents genes highly expressed in both groups. (c, d) GO terms of specific highly expressed genes in IFN-λ and IFN-α groups of SUS (c) and BGC (d). Red color highlights GO terms in the IFN-λ group that are mainly related to response to viruses, while yellow color indicates GO terms in the IFN-α group that are mainly related to regulation of response to wounding and cell death. The full names of GO terms with "*" in **(c)** were "Class I MHC-mediated antigen processing and presentation" and "Antiviral mechanism by IFN-stimulated genes." (e, f) Dot plot showing ISG expression in SUS (e) and BGC (f). Dot color represents the expression level. Dot size represents the expression percentage. (g, h) Multiplex immunofluorescence staining of olfactory epithelial sections showing that Mx1 (red) expression differs between IFN-α, IFN-λ, and mock groups in SUS (g) and BGC (h). CK18 (green) was used as a marker for SUS. SOX9 (yellow) was used as a marker for BGC. DAPI (blue) was used to stain nuclei. Results are representative of three samples and shown as mean ± SEM. *P < 0.05, **P < 0.01, and ***P < 0.001, by one-way ANOVA with Tukey’s multiple-comparison test. NS, no significant difference. The white bar represents 30 µm.

Recent studies demonstrated that SARS-CoV-2 primarily infects SUS and BGC but not olfactory sensory neurons in the olfactory epithelium, resulting in anosmia in COVID-19 patients (19, 21, 23). When evaluating differentially expressed genes, we noticed that IFN-λ triggered a stronger response regulating a larger gene set in SUS and BGC compared to IFN-α (Fig. 3a and b). In SUS and BGC, IFN-λ upregulated more genes related to antiviral immunity, including genes involved in “antigen processing and presentation,” “response to virus,” and “class I MHC mediated antigen processing and presentation” (Fig. 3c and d). Furthermore, some canonical ISGs (*Mx1*, *Isg15*, *Ifit1*, *Ifit3*, *Rsad2*, *Oasl2*, *Stat1*, Stat2, *Ifi44*, *Ifit3b*, *Irgm1*, and *Gbp7*) were more dominantly upregulated by IFN-λ than IFN-α in SUS and BGC (Fig. 3e and f). The high degree of responsiveness of SUS and BGC to IFN-λ was confirmed by immunofluorescence staining of URT tissue sections. This analysis showed that the ISG MX1 was readily detectable in the nuclei of SUS and BGC in IFN-λ-treated mice (Fig. 3g and h). Collectively, these results suggested that IFN-λ might confer robust antiviral protection of SUS and BGC.

### IFN-λ protects the olfactory epithelium against SARS-CoV-2

**Fig 4:**
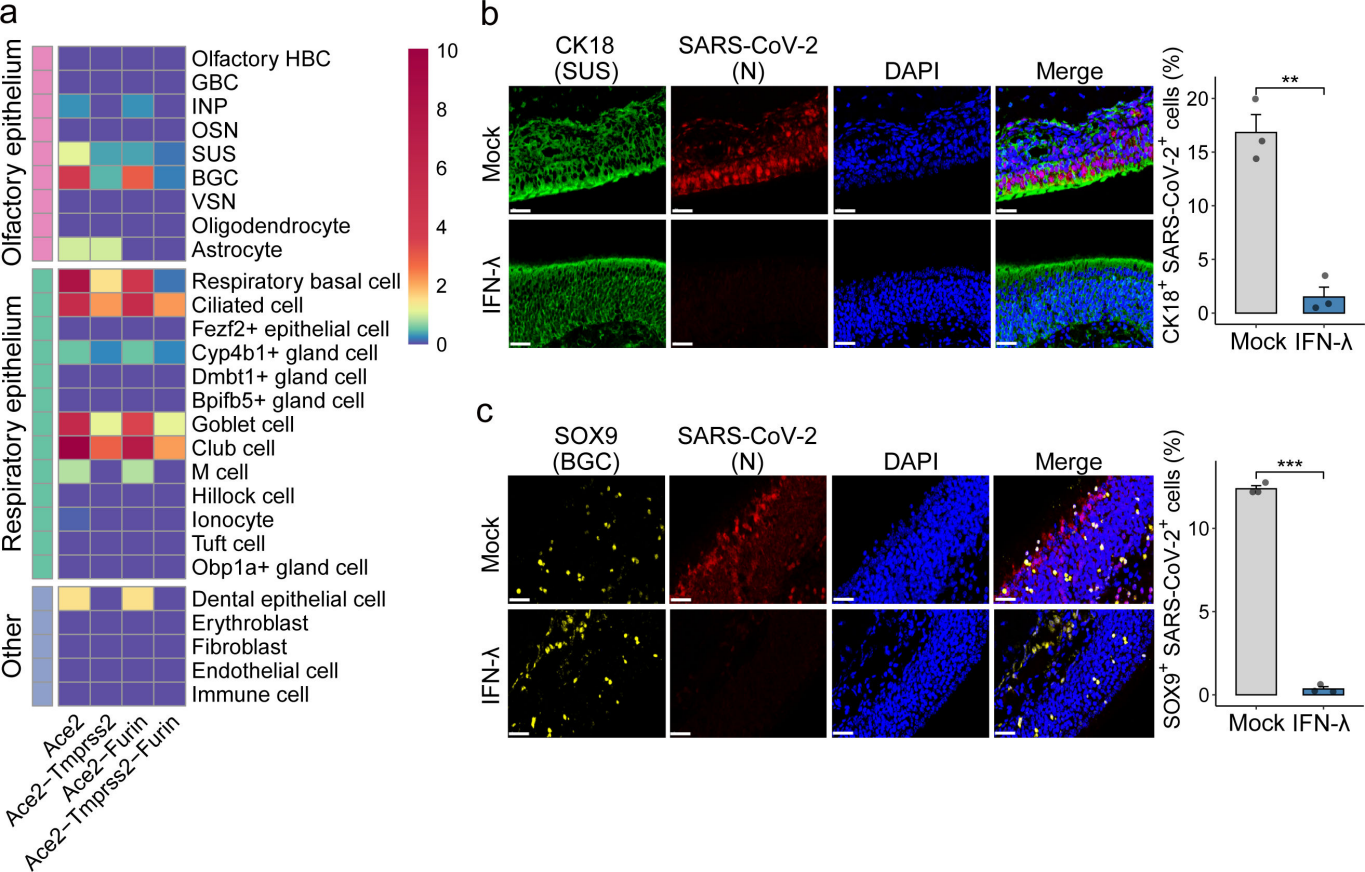
IFN-λ treatment prevents SARS-CoV-2 infection of the olfactory epithelium. (a) Co-expression percentage of *Ace2*, *Tmprss2*, and *Furin* in different cell types of untreated mice. (b, c) Multiplex immunofluorescence staining of sections of the olfactory epithelium showing SARS-CoV-2 replication in SUS (b) and BGC (c) in mock and IFN-λ-treated mice. CK18 (green) was used as a marker for SUS. SOX9 (yellow) was used as a marker for BGC. SARS-CoV-2 (red) was detected by staining for the viral N protein. DAPI (blue) was used to stain nuclei. Results are representative of three samples and shown as mean ± SEM. **P < 0.01; ***P < 0.001, by unpaired two-tailed Student’s *t*-test. The white bar represents 30 µm.

SARS-CoV-2 enters the host cell via interactions between the viral spike protein and cell surface-exposed angiotensin-converting enzyme 2 (ACE2). Virus maturation and successful entry further depend on cellular proteases, including furin, transmembrane serine protease 2 (TMPRSS2), and other proteases (14, 15, 30–32). Recent studies reported that *ACE2* and *TMPRSS2* are abundantly expressed in the human upper respiratory epithelium, including ciliated, goblet, and club cells (19, 26, 33). To determine which epithelial cell types in the upper airways of mice express *Ace2*, *Tmprss2*, and *Furin*, we screened our data for co-expression of these viral maturation and entry mediators in the respiratory and olfactory epithelium. We found that many respiratory epithelial cell subtypes, including RBCs, ciliated cells, Cyp4b1^+^ gland cells, goblet cells, and club cells, co-expressed *Ace2*, *Tmprss2*, and *Furin* (Fig. 4a; Fig. S5). In the olfactory epithelium, SUS and BGC were the cell types that most frequently co-expressed *Ace2*, *Tmprss2*, and *Furin* (Fig. 4a; Fig. S5), suggesting that SUS and BGC in the upper respiratory tract of mice are highly susceptible to SARS-CoV-2 infection.

IFN-λ provides effective antiviral protection in UTR, preventing the spread of Influenza A and Sendai viruses to the lungs and limiting viral transmission (4). To directly verify the antiviral potential of IFN-λ in the olfactory epithelium, we treated mice with either buffer solution or with a chimeric version of human IFN-λ drug candidate before infection with a mouse-adapted SARS-CoV-2 strain (MA20) (34, 35). Two days later, the animals were sacrificed, and immunofluorescence analysis of URT sections was performed. Using antibodies directed against the nucleoprotein of SARS-CoV-2, we observed a large number of virus-positive cells in the olfactory epithelium of buffer-treated mice (Fig. 4b and c). Some of the infected cells were CK18- or SOX9-positive, identifying them as SUS and BGC (19, 21), respectively (Fig. 4b and c). By contrast, in URT sections of IFN-λ-treated mice, we observed no virus-specific signals, indicating that SARS-CoV-2 replication was efficiently blocked by the IFN-λ treatment (Fig. 4b and c). These results demonstrated that IFN-λ is highly effective in preventing SARS-CoV-2 infection of SUS and BGC as well as other virus-susceptible cells in the olfactory epithelium, emphasizing its potential to prevent virus-mediated olfactory disorders.

## DISCUSSION

Previous work demonstrated that type I and type III IFNs have nonredundant antiviral functions at mucosal barriers (3–5). For example, IFN-λ proved to be more efficient than IFN-α in controlling respiratory virus spread from the upper airways to the lungs, and it was more potent in reducing virus transmission to new hosts (4). The molecular basis of this functional heterogeneity is still incompletely understood. A study using a reporter mouse indicated that the olfactory epithelium is enriched in IFN-λ receptor-expressing cells (36), but the functional consequence of this observation remained unclear. In this study, we used an unbiased single-cell transcriptome profiling approach to generate a comprehensive cell atlas of the respiratory and olfactory epithelium. This approach revealed striking sensitivity differences of individual epithelial cell subtypes to IFN-λ and IFN-α. IFN-λ acted preferentially on cell types forming the olfactory epithelium, especially SUS and BGC, whereas IFN-α preferentially regulated gene transcription in the respiratory epithelium. Our observation that cell type-specific sensitivity to IFN-α and IFN-λ in the upper airways is more heterogeneous than previously appreciated supports the concept that the two IFN subtypes have nonredundant functions, and it further shows that the antiviral defense of the epithelium lining the nasal cavity is compartmentalized.

Interestingly, although olfactory epithelial cells responded significantly stronger to stimulation with IFN-λ, they still responded reasonably well to IFN-α. However, IFN-λ-mediated differential regulation of a larger gene set compared to IFN-α, and IFN-λ preferentially regulated the expression of genes involved in antiviral defense, indicating both quantitative and qualitative differences in the respective IFN-mediated responses. Since the signaling pathways downstream of the type I and type III IFN receptors are remarkably similar, this observation was rather unexpected. Only few recent studies demonstrated differences in type I and type III IFN signaling pathways with regard to their dependency on receptor-associated signaling molecules such as TYK2 (37, 38), and only few studies showed that different cell types can respond differently to type I and type III IFN (5, 37). In primary mouse tracheal epithelial cells, type I and III IFN induced a highly overlapping transcriptional response, whereas in neutrophils, type I IFN additionally upregulated the expression levels of genes involved in inflammation. Furthermore, in contrast to epithelial cells, type III IFN failed to induce the expression of some canonical ISGs such as *Mx1* in neutrophils (5, 37). Additional studies are required to better understand IFN signaling integration in highly differentiated cell types with specialized functions.

The olfactory epithelium is a key site for SARS-CoV-2 infection in humans, and infection of SUS and BGC in the upper airways may result in olfactory dysfunction (20, 21, 39–41). In some COVID-19 cases, olfactory impairment is long term and most likely permanent, and loss of olfactory function has profound impacts on nutrition as well as physical and psychological health (42). Currently, there is no effective therapy for COVID-19 olfactory dysfunction (42, 43). IFN-λ was recently identified as a potential therapeutic drug to inhibit SARS-CoV-2 infection in the upper respiratory tract and lungs (35). Our data with the mouse model demonstrated that administration of IFN-λ prevents SARS-CoV-2 infection of SUS and BGC, highlighting the potential use of this antiviral cytokine as a therapeutic drug to prevent virus-induced olfactory disorders. IFN-λ treatment protects against respiratory viruses without substantial side effects (44). However, further research is needed to clarify this point before conducting preclinical trials with IFN-λ to prevent virus-induced olfactory impairment.

## MATERIALS AND METHODS

### Animals

Eight-week-old C57BL/6 female mice (which are commercially available) were used for the scRNA-seq work to reduce breeding work. These animals were purchased from the Jackson Laboratory. Eight-week-old B6.A2G-Mx1 mice (which carry functional Mx1 alleles) were used for *in situ* staining and infection experiments to facilitate monitoring the antiviral effects of IFNs (7, 45). All mice were kept in a temperature- and humidity-controlled animal facility under specific pathogen-free conditions in the local animal facility.

### Mouse snout cell dissociation

Groups of mice (*n* = 15) were treated intranasally with either 1 µg of human IFN-αB/D (referred to as IFN-α, ref (46).) or 1 µg mouse IFN-λ2 (referred to as IFN-λ, ref (47).), which was the effective dose for activating epithelial cells reported previously (7), in a 30 µL volume for 5 hours, before the animals were sacrificed. Snout tissue from each group was collected and mechanically disrupted before five cycles of trypsin (1  mg/mL) digestion at 37  °C on a thermoshaker for 7–10 minutes each were applied. The released cells were transferred to DMEM (Gibco, Life Technologies) containing 10% fetal calf serum (FCS) and were then passed through a 70-µm cell strainer. After removing red blood cells using a red blood cell lysis buffer (0.15 mM NH_4_Cl, 10 mM KHCO_3_, and 0.1 mM EDTA-Na_2_ in Milli-Q water) on ice for 3 minutes, the cells were washed in FACS buffer (1% BSA, 0.1% NaN_3_ in PBS) and used for epithelial cell purification.

### Snout epithelial cell purification and flow cytometry analysis

Single-cell suspensions derived from snouts were depleted of CD45^+^ using Mojosort mouse CD45 nanobeads (BioLegend, 480028) and magnets according to the manufacturer’s instructions. In brief, snout single cells were washed with MojoSort buffer and incubated for 15 minutes on ice with mouse CD45 nanobeads. After incubation, the beads were washed with MojoSort buffer, centrifuged at 1,600 rpm for 5 minutes, resuspended in 2.5 mL MojoSort buffer in polypropylene tubes, and placed in the magnetic column for 15 minutes at 4°C. The cells in the CD45-depleted fraction were analyzed for epithelial cell purity by flow cytometry using anti-CD45 (BioLegend, 30-F11) and anti-EpCAM (BioLegend, G8.8). Mouse snout epithelial cell suspensions of sufficient purity (> 98%) were used to prepare scRNA-seq libraries.

### scRNA-seq library preparation

The prepared single-cell suspension was processed for scRNA-seq using 10 x Genomics Single Cell 3’ Library Construction Kit v3 (10 x Genomics, Pleasanton, CA) following the manufacturer’s protocols. Approximately 2.6 × 10^4^ cells for mouse samples were loaded across each run of the chip to produce the Gel Beads-in-Emulsions (GEMs), followed by reverse transcription, cDNA amplification, and library construction. Qubit and Qsep100 were used to detect the library concentration and product length before sequencing, respectively. The library was sequenced on the Illumina NovaSeq6000 instrument with 150-bp paired-end reads. The library structure contained 28 bp Read1, 8 bp I7 Index, 8 bp I5 Index, and 91 bp Read2.

### Data analysis

#### Generation of scRNA-seq matrix

Cellranger 5.0.0 (10x Genomics) was utilized to perform demultiplexing, map reads to the reference genome (refdata-cellranger-mm10-3.0.0), correct cell barcode/UMIs, perform expression counting, and generate a single-cell expression matrix. Subsequently, the raw matrix was loaded into Seurat 4.1.1 (48) for further analysis.

#### Quality control

Rigorous quality control was implemented on the raw data. The expression matrix was loaded, and top 50 PCs were employed for cell clustering. A relatively high percentage of mitochondrial genes was observed. Prior to filtering cells based on the mitochondrial content, an initial filtering step was applied to remove low-quality cells, defined as clusters with a median gene lower than 100. Some of these clusters exhibited mitochondrial gene percentages exceeding 70%. After this initial filtering, cells with more than 50% mitochondrial gene expression were defined as outliers and subsequently removed from the analysis. To identify and remove potential doublets, python package scrublet (49) was applied with default pipeline and parameter “n_prin_comps = 50.” Since the distribution of doublet scores was bimodal distribution, the lowest point between the two peaks of bimodal distribution is considered the threshold. Cells surpassing this threshold were considered doublets and would be removed.

#### Clustering

The expression matrices of mice samples were merged together. Data were normalized using the *LogNormalize* algorithm, and 3,000 high variable genes were identified using the *vst* algorithm. We standardized expression values for each gene across all cells using z-score transformation. Unsupervised linear dimensionality reduction was performed via PCA on the data’s top 50 PCs, utilizing a shared nearest neighbor (SNN) algorithm with parameters set at 30 perplexities. We finally used a resolution of 1.5 to identify cell clusters of mouse nasal epithelium. Immune cells (*Ptprc*) and erythrocytes (*Hbb-bs1* and *Hba-a1*) were removed based on its specific gene expression. All the functions mentioned here were from R package Seurat 4.1.1 (48).

#### Data visualization

Uniform manifold approximation and projection (UMAP) (50, 51) was employed for nonlinear dimension reduction, ensuring computational efficiency. The PCs used for cell clustering were also utilized to calculate cell embeddings.

#### Calculation of effect score

Effect score was utilized to evaluate the impact of IFN-α and IFN-λ treatment on distinct cells, which was calculated based on information entropy. For each cell, the proportion of the nearest 20 cells (include itself) within each group (Mock, IFN-λ, and IFN-α groups) was used to calculate neighborhood entropy (NE). The NE was calculated through the formula:


NE= −log2(P(λ))∗P(λ)− log2(P(α))∗P(α)− log2(P(M))∗P(M)

where *P(λ*), *P(α),* and *P(M)* represent the cell distribution probability of the nearest 20 cells in IFN-λ, IFN-α, and mock groups, respectively, and the effect score was designed as NE subtracted by its maximum value:


 Effect score=Max(NE)−NE

NE and effect score for each cell can be calculated. The distribution of the 20 nearest neighboring cells across the three groups becomes more uneven, resulting in a lower NE score and a higher effect score, suggesting that IFN-α and IFN-λ treatments have a greater impact on the cells.

#### Identification of differentially expressed genes

Differentially expressed genes (DEGs) between two cell groups were identified using *FindAllMarkers* or *FindMarkers*, in which the Wilcoxon test (two-sided) was set as default. Bonferroni test was used for multiple comparison correction. Cluster-specific expressed genes were extracted using R package dplyr 1.1.4 (52).

#### GO enrichment analysis for differentially expressed genes

GO enrichment analysis of the differentially expressed genes was performed by Metascape, which integrated multiple databases and was a user-friendly web-based portal (https://metascape.org/) (53). The results were visualized as heatmap or bar plot using the R package ggplot2 (54).

#### Gene set enrichment analysis (GSEA)

R package clusterProfiler (55) was employed for GSEA on specific GO terms. Initially, differentially expressed genes between IFN-λ and IFN-α groups were extracted and ranked according to log2(FC) value. Then, *gseGO* function was applied with parameters “ont=’BP’, pvalueCutoff = 0.05, pAdjustMethod=’BH’.” Finally, *gseaNb* function was used to present the GSEA results.

#### Calculation of ISG score

AUCell (50) was employed to calculate the ISG score for each single cell. The 60 canonical ISGs (*Irf1, Irf3, Irf7, Irf9, Stat1, Stat2, Sp100, Socs1, Usp18, Mx1, Mx2, Isg15, Isg20, Oas1g, Oas1a, Oas2, Oas3, Oasl1, Oasl2, Eif2ak2, Ifit1, Ifit2, Ifit3, Ifitm1, Ifitm2, Ifitm3, Ifi35, Ifi6, Ifi27, Ifi30, Ylpm1, Ddx58, Ddx60, Rsad2, Mov10, Ifih1, Hpse, Gbp2b, Gbp2, Gbp5, Ifi44l, Trim5, Trim12c, Trim25, Trim32, Trim69, Parp12, Parp14, Ch25h, Zcchc3, Znfx1, Rbbp6, Ido1, Cxcl10, H2-K1, H2-D1, H2-Q4, H2-Q6, H2-Q7, H2-Q10, H2-Q1, H2-Q2, H2-T23, Pml,* and *Samhd1*) were collected from the Gene Ontology database and were used for ISG score calculation. Area under the curve (AUC) was adopted in AUCell to calculate whether the ISG gene list is enriched in each cell. The *AUCell_buildRankings* function was employed to rank gene expression using normalized data. The *AUCell_calcAUC* function was utilized to calculate the AUC for the ISG gene set in each cell. The results were extracted using the *getAUC* function and visualized in box plots.

### Virus infection and multiplex immunofluorescence staining

B6.A2G-Mx1 mice were subcutaneously treated with 3 µg IFN-α or 3 µg hIFN-λ1/3 (34, 35), which was the effective antiviral dose reported previously (35), in a 100 µL volume on days −1, 0, and 1 post-infection. Mice were anesthetized using 1.8%–2.8% isoflurane in O_2_ and intranasally inoculated with 10^3^ PFU of a mouse-adapted SARS-CoV-2 strain (MA20) (35) in a 40 µL volume. On day 2 post-infection, mice were anaesthetized with ketamine/xylazine, perfused with 10% formalin via the left ventricle, and the tissue was stored in 10% formalin until further processing. Snouts were embedded as formalin-fixed paraffin-embedded (FFPE) blocks for multiplex immunofluorescence (mIF) staining.

The mouse snout FFPE blocks were cut into 4-µm sections by a rotary microtome (Leica RM2255, Germany). The sections were deparaffinized, rehydrated, subjected to heat-induced epitope retrieval, and incubated with primary and secondary antibodies. The antibodies were visualized using fluorescent tyramide of the Opal Detection Kit (NEL861001KT, Akoya Biosciences). The process of epitope retrieval and staining was repeated sequentially for different primary antibodies and fluorescent tyramide combinations. The following primary antibodies with different dilutions were used: SOX9 (ab185230 Abcam) with 1:50 dilution, cytokeratin 18 (CK18, #10830–1-AP, Proteintech) with 9 µg/mL, Mx1 (anti-AP5 peptide) (45) with 100 µg/mL, and SARS-CoV-2 nucleocapsid protein (#26369, Cell Signaling) with 1:50 dilution. Goat anti-rabbit IgG H&L (ab214880, Abcam) was used as a secondary antibody. Antibodies were visualized with the following tyramide dyes used from the Opal Detection Kit (NEL861001KT, Akoya Biosciences): Opal 480, Opal 520, Opal 570, and Opal 620. DAPI was used for cell nuclear staining. Sections were mounted with ProLong Diamond Antifade Mountant (P36961, Thermo Fischer Scientific). Images were scanned using the PhenoImager Fusion system (Akoya Biosciences) and further analyzed using QuPath to calculate the percentage of positive cells.

### Statistical analyses

The comparison of data between different groups was done by the Wilcoxon test (two-tailed), one-way ANOVA with Tukey’s multiple-comparison test, or unpaired two-tailed Student’s *t*-test. *P* values < 0.05 were considered significant. Levels of significance are indicated as follows: ^*^P < 0.05, ^**^P < 0.01, and ^***^P < 0.001. The statistical analyses were conducted in R (v 4.2.0). Most figures in this study were plotted using the ggplot2 package.

## Data Availability

All the data needed to evaluate the conclusions are available in the article and supplemental material. The scRNA-seq data employed in this work were deposited in the Genome Sequence Archive in the National Genomics Data Center, China National Center for Bioinformation/Beijing Institute of Genomics, Chinese Academy of Sciences, under accession number CRA015666. Additionally, the relevant codes are publicly available on github at https://github.com/snow55/IFN_lambda_in_OlfactoryEpi/tree/master.

## SUPPLEMENTAL MATERIAL

Supplemental figures jvi.01413-24-s0001.docx Figures S1 to S5.

ASM does not own the copyrights to Supplemental Material that may be linked to, or accessed through, an article. The authors have granted ASM a non-exclusive, world-wide license to publish the Supplemental Material files. Please contact the corresponding author directly for reuse.
