# Supplementary material for: Single-cell transcriptomics reveals a compartmentalized antiviral interferon response in the nasal epithelium of mice
Source: J Virol. 2025 Feb 4;99(3):e01413-24. doi: 10.1128/jvi.01413-24 (PMC11915831; doi:10.1128/jvi.01413-24)
Supplement: Supplemental figures — Figures S1 to S5. [file jvi.01413-24-s0001.docx]

**Supplementary figure and figure Legends**

**
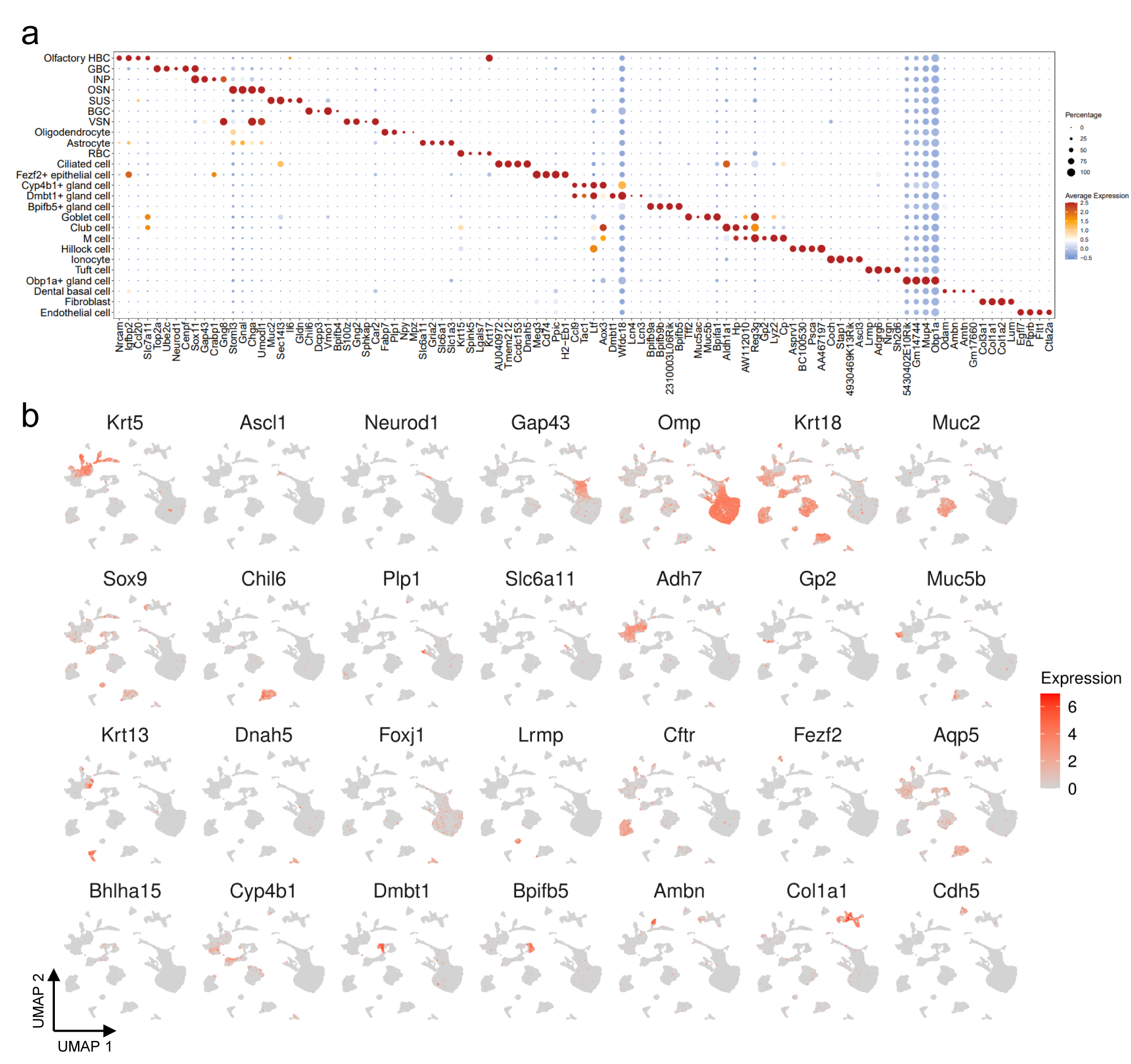
**

**Figure S1.** **The marker genes for each type of cell within the mouse nasal epithelium. (a)** Dot plot showing the top four differentially expressed genes of each cell type in the mouse nasal epithelium. Dot color represents expression level. Dot size represents expression percentage. **(b)** UMAP visualization of cell type-specific marker genes in the mouse nasal epithelium.
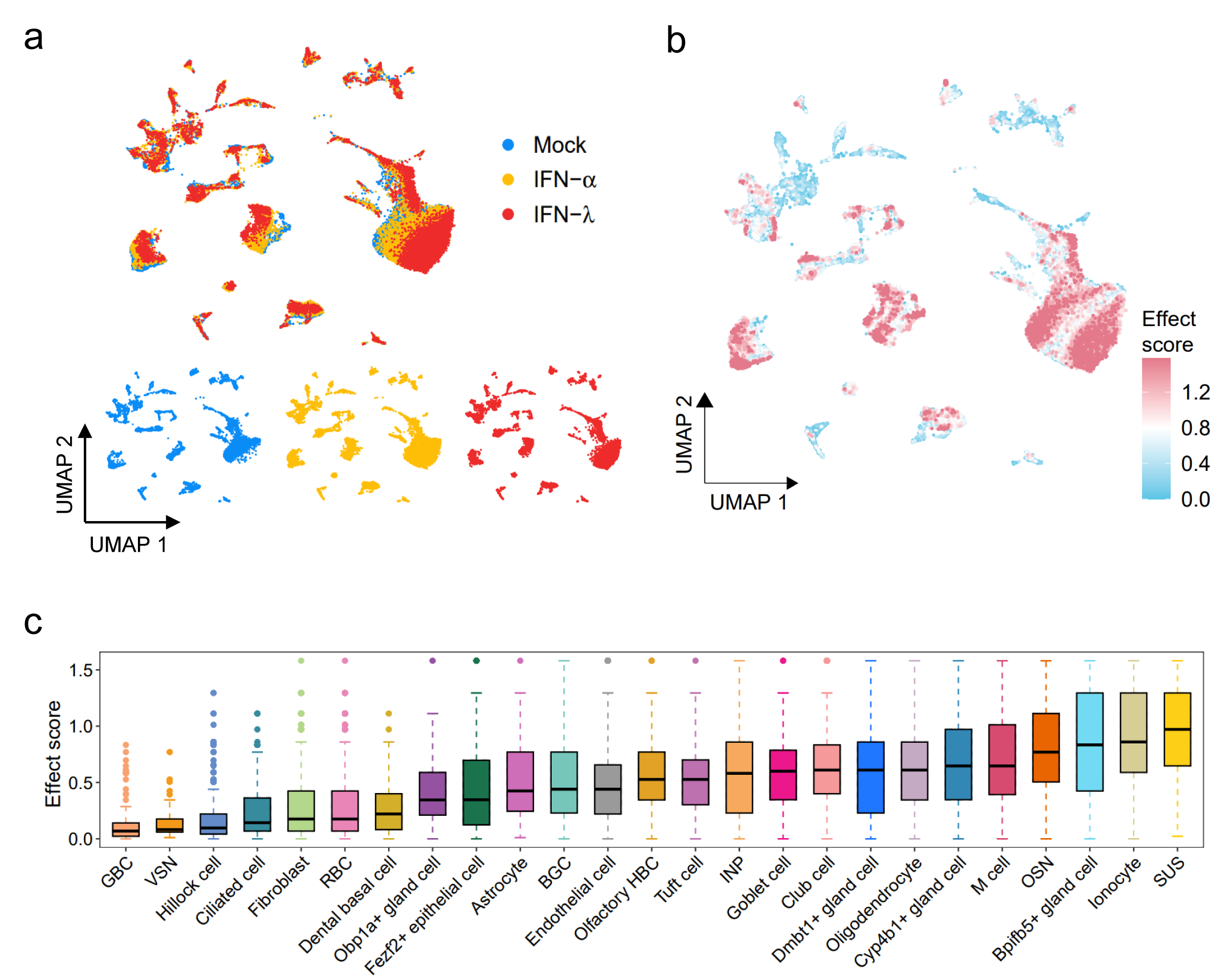


**Figure S2. Heterogeneity of the mouse nasal epithelium in response to IFN-α or IFN-λ. (a)** UMAP visualization of all cells (upper panel) and split UMAP plots (lower panel) of cells from mock, IFN-α, or IFN-λ treatment groups. Cells were colored by group. **(b)** UMAP visualization of all cells from three groups colored by the effect score (see **Methods**). **(c)** Box plot illustrates the effect score of each cell type. The higher effect score, the stronger impact of IFN-α and IFN-λ treatments on cell types.**
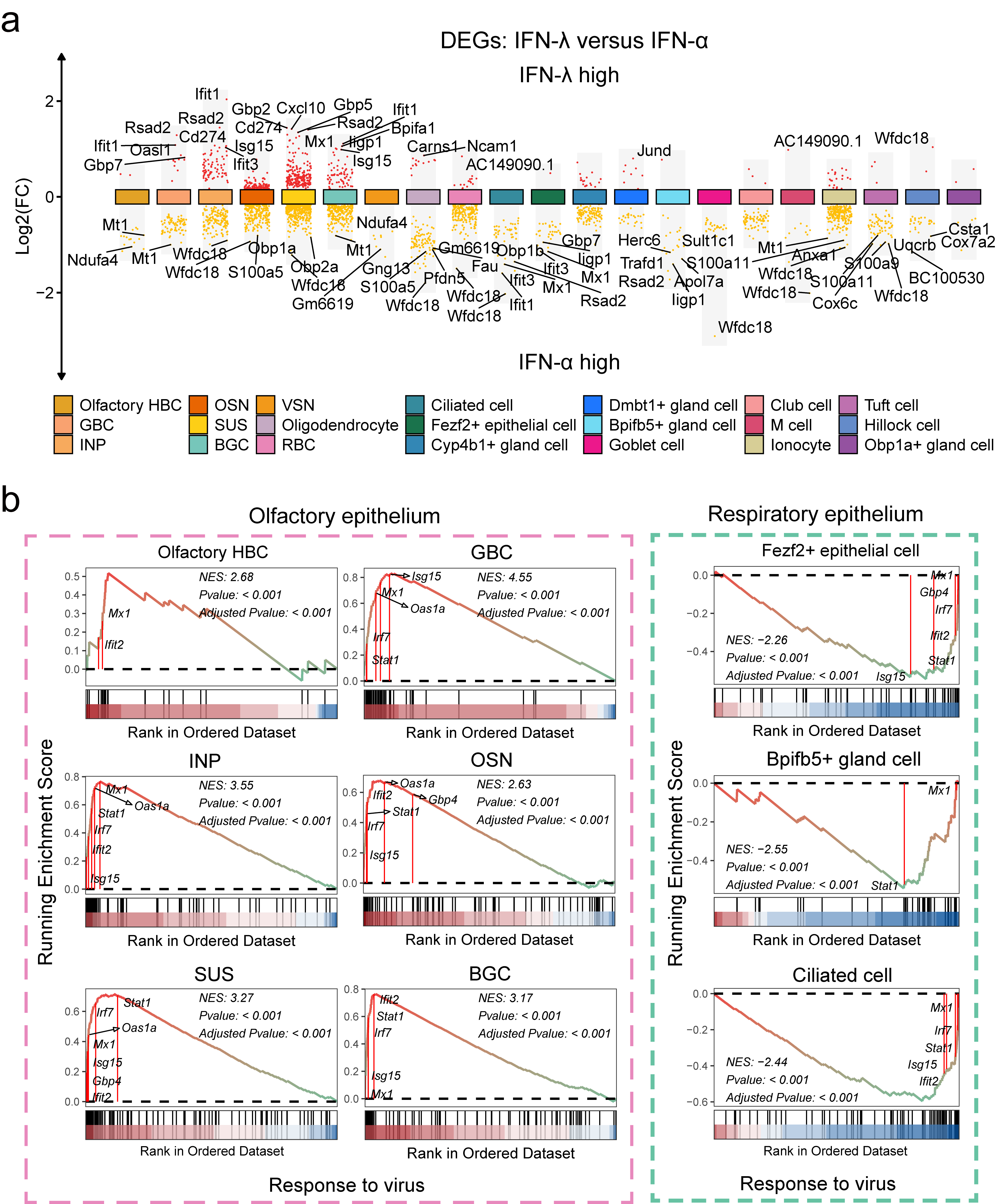
**

**Figure S3. Heterogenous antiviral response in the mouse nasal epithelium triggered by IFN-α and IFN-λ. (a)** Differentially expressed genes (DEGs) between the IFN-α and IFN-λ groups in all cell types (adjusted P value < 0.05, Bonferroni method). Red and yellow points represent up-regulated genes in IFN-λ and IFN-α treated mice, respectively. **(b)** DEGs from IFN-λ versus IFN-α in each cell type were used to conduct GSEA analysis. Gene rank plot of GSEA resulting from the term “response to virus” in Olfactory HBC, GBC, INP, OSN, SUS, BGC, Fezf2^+^ epithelial cells, Bpifb5^+^ gland cells and Ciliated cells. NES, normalized enrichment score. NES > 0 represents enrichment in the IFN-λ group, while NES < 0 represents enrichment in the IFN-α group.


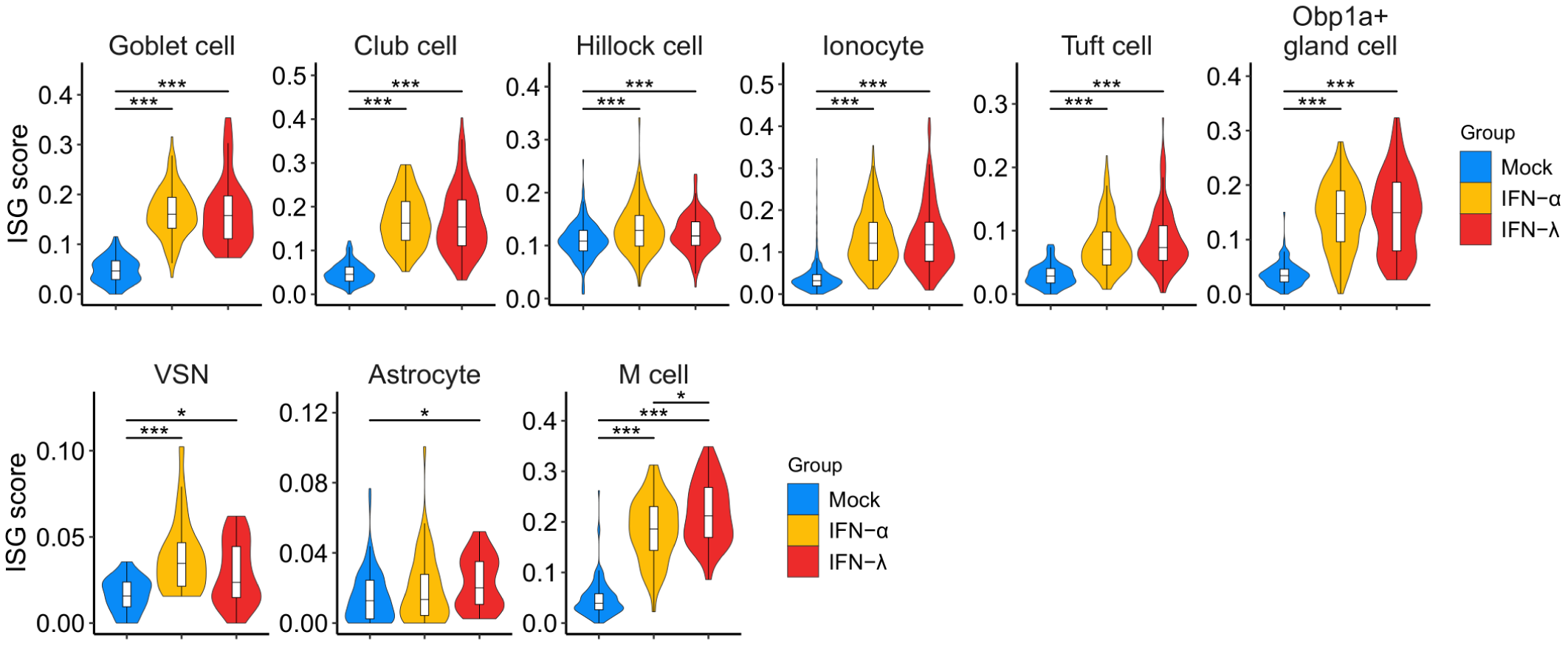


**Figure S4. Cell type-specific IFN-α and IFN-λ induced ISG scores in the nasal epithelium.** Violin plot of the ISG score in various cell types divided into three groups. The ISG score was calculated by the AUCell package based on 60 canonical ISGs. The statistical differences in the ISG score among three groups were calculated by the Wilcoxon test (two-sided). *P < 0.05; **P < 0.01; ***P < 0.001.


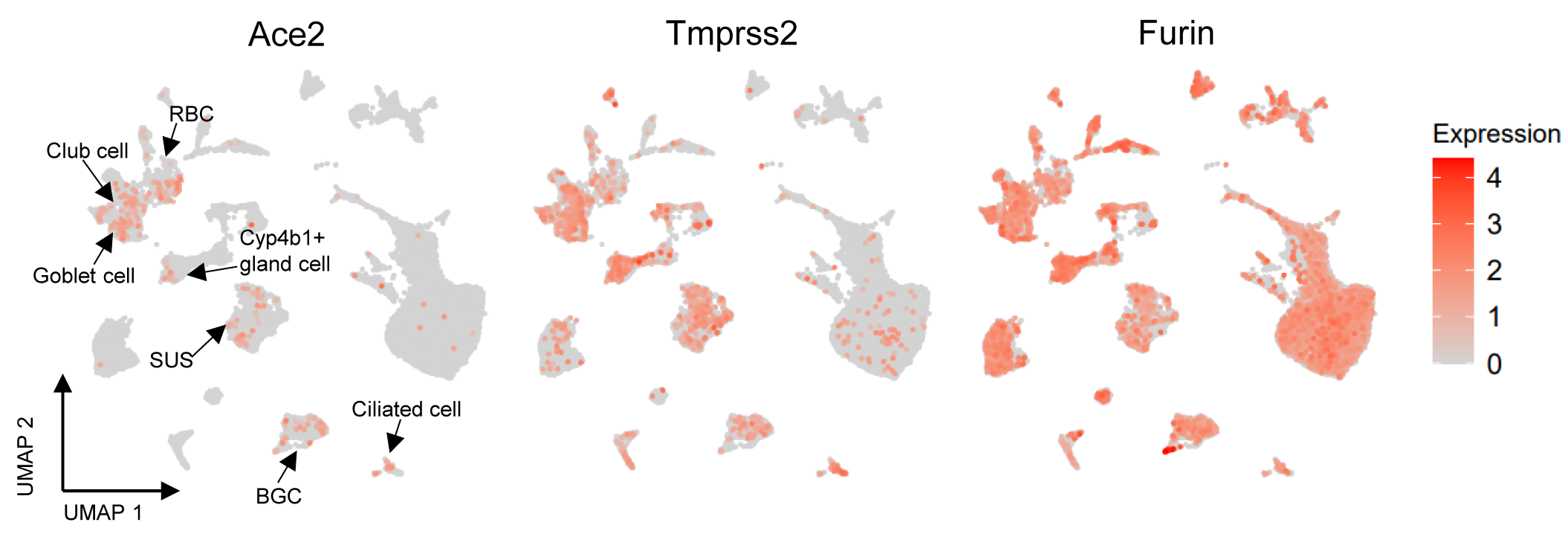


**Figure S5. Cell type-specific expression pattern of host-encoded SARS-CoV-2 maturation and entry factors.** UAMP visualization of *Ace2*, *Tmprss2*, and *Furin* expression in all cell types. Color represents expression level.
